# A proposed framework for holding intensive 3Rs workshops in laboratory animal science

**DOI:** 10.1186/s42826-022-00120-9

**Published:** 2022-03-29

**Authors:** Siavash Ahmadi-Noorbakhsh, Jila Sadighi, Zahra Hatami, Ehsan Shamsi Gooshki

**Affiliations:** 1 National Committee for Ethics in Biomedical Research, Ministry of Health and Medical Education, Tehran, Iran; 2 Comparative Medicine Group, Preclinical Core Facility, Tehran University of Medical Sciences, Tehran, Iran; 3 Department of Health Promotion, Health Metrics Research Center, Institute for Health Sciences Research, ACECR, Tehran, Iran; 4 Medical Ethics and History of Medicine Research Center, Department of Medical Ethics, Faculty of Medicine, Tehran University of Medical Sciences, Tehran, Iran

**Keywords:** Laboratory animals’ workshop, Workshop, Short-course, 3Rs, Biomedical workshop, Bio methodology workshop, Training, Education, Teaching

## Abstract

Principles of 3Rs are the backbone of today’s animal research. Applying 3Rs in practice requires proper education and training. Although this could be achieved via long-term courses ranging from several weeks to years, in some circumstances a short-term course may be the only viable option. In this paper we define scientific and ethical objectives for running short-term 3Rs workshops. To meet these objectives, we propose a 2-day workshop curriculum and an ethical framework. The curriculum comprises theoretical and practical sessions and covers Replacement, Reduction, and Refinement strategies. The ethical framework defines the responsibilities of lecturers and attendees, and proposes the animals and facilities requirements to run the proposed workshop curriculum. The attendees will be assessed at the end of the workshop and they receive certificates for working under supervision until they are deemed competent at their intended functions. The proposed curriculum and framework are not subscriptive, instead they share the experience gained through running more than 60 laboratory animal 3Rs workshops.

**Supplementary Information:**

The online version contains supplementary material available at 10.1186/s42826-022-00120-9.

## Background

The proper care and use of laboratory animals are best described by 3Rs principles that emphasize Replacing, Reducing, and Refining the use of animals for scientific purposes [1]. Adhering to the 3Rs principles requires a fair amount of theoretical knowledge and practical skills. Ideally these may be achieved by enrolling in a three-year residency program in laboratory animal medicine by graduated veterinarians [2]. Alternatively, one could attend short-term courses according to his/her function [3]. The American Association for Laboratory Animal Science also provides certification programs for laboratory animal technicians and technologists [4]. These courses may be completed within a semester or a few weeks and there are also educational materials available for self-study [4].

However, research shows that there are unmet educational and training needs among animal research workers [5]. In fact, in some circumstances a short workshop may be the only viable option. This may be true of faculty members, postgraduate students, or animal care staff who only need to perform basic procedures on animals and are not suitable candidates for longer courses. Also, researchers who need to practice and refine their skills may benefit from a short workshop. In a broader perspective, the care and use of laboratory animals are still in the developing stages in a considerable number of countries. The available infrastructures and financial resources in these countries may not be sufficient for running well-structured laboratory animal courses similar to Europe [3] or US [4, 6]. However, there are still a considerable number of biomedical researches undergoing in these countries, consuming a large number of laboratory animals per year. Therefore, there is a substantial need to properly address the educational and training requirements of the abovementioned audiences.

In this regard, we developed a framework for holding intensive two-day workshops. This framework is intended to assist attendees in acquisition of required knowledge and skills for properly and ethically performing supervised laboratory animal experiments. Additional training and supervised practice will be necessary to gain proficiency in the techniques for unsupervised work with animals. This workshop framework is not meant to be a prescriptive document. Instead, it is aimed to reflect the knowledge and experience gained through performance of a large number of previous workshops, and to incorporate this knowledge and experience into the planning and execution of future courses.

## Main text

### Workshop planning

For developing the workshop plan, we used the experience gained through holding more than 60 laboratory animal workshops in the country. We also adhered to the principles outlined in our Guideline for the Care and Use of Laboratory Animals [7]. We first defined the scientific and ethical objectives of the workshop. To define the scientific objectives, we consulted a relevant European commission guidance document [3]. According to these scientific objectives, at the end of this workshop the attendees should be able to:

- describe how 3Rs principles affect the scientific quality of animal researches,
- describe the alternative methods that could be used instead of animal testing in projects,
- interpret the basic principles of animal research methodology,
- summarize the biological characteristics of common species and strains of laboratory animals,
- describe proper standards of care and husbandry of animals in research settings,
- identify the principles of safety and security in working with laboratory animals and naming the commonest zoonotic diseases,
- interpret the principles of sampling or materials administration,
- interpret the methods of recognizing pain and distress in laboratory animals,
- differentiate anesthesia and analgesia and describe proper methods of anesthesia/analgesia in common laboratory animal surgeries,
- summarize humane endpoints, and define proper methods of euthanasia and management of unexpected animals’ deaths,
- demonstrate proper handling methods of mice and rats,
- perform proper sexing of animals,
- perform dose calculation, and demonstrate proper methods of administrating substances to animals, and
- apply proper techniques for blood sampling.

Then we developed an intensive 2-day workshop curriculum to achieve the scientific objectives via theoretical and practical sessions. This workshop curriculum is presented in Supplement 1 to this paper (see Additional file 1).

The ethical objectives of the workshop are set to enable attendees to:

- describe the basic ethical principles underlying the use of animals for scientific purposes, specifically the 3Rs concept;
- describe why we should adhere to the ethical treatment of laboratory animals;
- describe how animal welfare affects the scientific results of a study;
- describe how they could access and use the national “Guide for the Care and Use of Laboratory Animals”;
- describe how performing or not performing each action in an animal research setting may have ethical consequences;
- identify the ethical methods of performing the common techniques on laboratory animals; and
- apply the ethical principles of care and use of laboratory animals in practice; and the attendees should:
- be morally sensitized about the care and use of animals in research; and
- have experienced an ethical workshop in which the animals are treated with care and responsibility.

To achieve the ethical objectives, we developed an ethical framework through a 7-stage process as follows: (1) We defined four major components of a standard laboratory animal workshop as: animals; attendees; human resources (i.e. lecturer and supervisors); and facilities (i.e. venue, equipment, and materials). (2) We defined how each component would be exercised to achieve the scientific objectives of the workshop. (3) We explored how exercising each component may negatively affect the welfare of animals. (4) We applied the 3Rs principles for replacing the negatively affecting exercises where possible. (5) If full replacement was not possible, we defined strategies to reduce the number of those exercises and the number of affected animals. (6) We refined all the exercises involving animals so that they cause the least possible negative effects for the animals. (7) Finally, we combined the results of the stages 4–6 to develop the ethical framework. This framework is presented in Supplement 2 to this paper (see Additional file 2).

### Workshop execution

The workshops are delivered according to the workshop curriculum and the ethical framework. Certain strategies [8] should be undertaken to facilitate the adult learning. The first part of the workshop is dedicated to moral sensitization. In this regard, issues on animal rights and welfare are taught. Narrative stories about animals’ cognition and ability to feel pleasant and unpleasant feelings are used to empower the message and engage the attendees. In the next part, replacements to laboratory animals are discussed and methods of finding replacements are presented. An overview of the methodological principles of animal research is presented next, with emphasis on the methods of reduction of animal use. In the next part of the workshop, biological characteristics of common laboratory animal species are discussed. Safety measures in working with laboratory animals along with the most common zoonoses are presented next and the first theoretical part of the workshop is finished in the early afternoon of the first day.

The second part of the theoretical session is held in the morning of the second day, teaching methods of drug administration and sample collection. Methods of recognition of pain and distress are presented later, describing how to instantly detect signs of pain and distress, and methods for evaluating the animal welfare over time. Principles of anesthesia and analgesia are discussed next. The concluding section of the theoretical session describes humane endpoints and methods of euthanasia.

Attendees in the practical session are divided into groups of 15–20 and a practical session is held for each group. The practical sessions are arranged for the afternoons of the first and the second days. In the beginning of the practical session, an induction is delivered, attendees are divided into groups of five, a competent supervisor is assigned to each group, and lab record sheets as shown in Supplement 3 (see Additional file 3) are distributed. The practical session is held according to the order of the topics described in Table 1. For each topic, the theoretical instructions are taught first using slide presentation and video clips. Then complementary information about the technique is given by demonstration (where appropriate). Afterwards, the attendees practice the procedure under supervision of their group supervisor. The workshop lecturer rotates between groups, supervises group actions, and troubleshoots when needed. Various steps for holding the practical session, as presented in Table 1, are discussed in Supplement 4 to this paper (see Additional file 4).

**Table 1.** Order of the topics in the practical session. Mice techniques are taught first, rats and rabbits are discussed afterwards

	Topic	Method	Mice	Rats	Rabbit
1	Teaching normal behaviors of animals	SP, D	√	√	√
2	Practicing normal behaviors of animals	HT	√	√	×
3	Animals marking	HT	√	√	×
4	Teaching animal handling, sexing, restraining, and heterospecific play (only rats)	SP, D	√	√	√
5	Practicing animal handling, sexing, restraining, and heterospecific play (only rats)	HT	√	√	×
6	Giving rest and treats to animals	HT	√	√	√
7	Teaching dose calculation and effects of selected medications	WT	×	×	×
8	Teaching syringe handling	D	×	×	×
9	Practicing syringe handling on silicone egg shaped grip balls	HT	×	×	×
10	Teaching subcutaneous injection	SP	√	√	√
11	Teaching intraperitoneal injection	SP	√	√	×
12	Teaching intramuscular injection	SP	×	√	√
13	Practicing subcutaneous, intraperitoneal, and intramuscular (rats) injections using blunt needles	HT	√	√	×
14	Giving treats to animals	HT	√	√	√
15	Teaching oral gavage	SP	√	√	×
16	Teaching venous access techniques	SP	√	√	√
17	Practicing venous access techniques on injection pads	HT	×	×	×
18	Teaching simple techniques of managing anesthesia and analgesia	SP	√	×	×
19	Medication assignment to groups and dose calculation	HT	√	×	×
20	Anesthesia and analgesia given to two animals in each group	HT	√	×	×
21	Practicing the management of anesthesia and analgesia	HT	√	×	×
22	Practicing tail vein access	HT	√	×	×

D: Demonstration; HT: Hands-on Training; SP: Slide Presentation; WT: whiteboard teaching

### Workshop assessments

We suggest two types of assessments for the workshops: (1) assessment of the workshop holders, and (2) assessment of the workshop attendees. The workshop holders could be assessed from three viewpoints: executive aspects, scientific quality, and lecturer(s)’ performance. Some open-ended questions may also be considered to let the attendees freely express their opinions on various aspects of the workshop.

The executive aspects may involve 5-point Likert questions on the timing, place, and facilities of the workshop. The scientific quality assessment may involve questions on the effect of the workshop on enhancement of the knowledge and attitude of the attendees, ability of the workshop in resolving the work-related problems of the attendees, and appropriateness of the workshop plan with the educational needs of the attendees. The lecturers’ performance may be assessed from at least three viewpoints: discipline, teaching skills, and interaction with attendees.

Workshop attendees may be assessed from knowledge and attitude aspects. Ideally, the assessment should be performed pre- and post-workshop to evaluate the effectiveness of the workshop on enhancing their knowledge and attitude. At least one question should be derived from each theoretical topic and in accordance with the scientific objectives of the workshop. Attitude questions may cover a wide range of topics, such as animal feelings, sympathy with animals, the value of animals’ lives, replacing animals with non-animal alternatives, reduction of animals’ use, and refinement of procedures. These questions may be derived according to the ethical objectives of the workshop.

## Conclusions

The proposed workshop plan is developed to teach ethical and scientific aspects of the care and use of laboratory animals. The order and content of the theoretical session is based on the 3Rs principles. We first discuss the ethical aspects of using animals for scientific matters, aiming to morally sensitize the attendees. In this regard, we review ethics of animal use from various viewpoints: human conscience, religion, dialectics, societal concerns, scientific aspects, and law. By this, we aim to influence attendees having various viewpoints and attract their attention to the moral status of animals.

The concept of 3Rs shaped the whole structure of the workshop. By discussing the Replacements to laboratory animals, we emphasize on the 1st R. Afterwards, the 2nd R is covered by teaching how various experimental designs and methods of sample size calculation could lead to more reliable data and less use of animals. The rest of the workshop discusses the 3rd R from various viewpoints.

In the proposed workshop plan, no animal is used for ‘educational’ purposes and their use are only limited to training purposes. We recommend using surplus stock animals and animals from completed studies, that had been fully recovered from prior interventions with a severity score of mild to moderate [7]. The severity score of the proposed workshop plan is ‘mild’ and we use anesthetic/analgesic drugs for all animals undergoing painful interventions. Also, by using lab record sheets, we limit the cumulative discomfort resulting from the performance of multiple interventions on each animal. These provisions are in accordance with the European framework for education and training in laboratory animal science [3].

In comparison to the European framework for education and training in laboratory animal science [3], our proposed workshop framework covers the requirements of function A. It has the contents of the Core module and function A’s specific module with the addition of five more topics as safety procedures in the use of laboratory animals, alternatives to laboratory animals, basic topics in laboratory animal research methodology, principles of sampling and drug administration, and basic topics in anesthesia and analgesia. Since mere training does not deliver competence [3], the attendees need to work under supervision following completion of the workshop until they are deemed competent in the tasks they do.

The proposed workshop curriculum and ethical framework are provided as suggestions and are not subscriptive. We believe that they provide essential scientific and ethical structures for holding intensive 3Rs workshops in laboratory animal science.

## Supplementary Information

**Additional file 1:** Proposed workshop curriculum. Proposed workshop curriculum is presented in this supplement.

**Additional file 2:** Ethical framework for holding intensive laboratory animal workshops. Proposed ethical framework is presented in this supplement.

**Additional file 3:** Lab record sheet. A sample of the lab record sheet is presented in this supplement.

**Additional file 4:** Practical session structure. The structure of the practical session is presented in this supplement.

## Data Availability

All relevant data are presented in the article.
