# Supplementary material for: A proposed framework for holding intensive 3Rs workshops in laboratory animal science
Source: Lab Anim Res. 2022 Mar 29;38:10. doi: 10.1186/s42826-022-00120-9 (PMC8966152; doi:10.1186/s42826-022-00120-9)
Supplement: Supplementary file 1 — Additional file 1: Proposed workshop curriculum. Proposed workshop curriculum is presented in this supplement. [file 42826_2022_120_MOESM1_ESM.docx]

**Proposed workshop curriculum**

| **Day 1** | |
| --- | --- |
| **Time** | **Topic** |
| 8:15 – 8:30 | Workshop registration |
| 8:30 - 8:45 | Completing the pre-workshop questionnaires |
| 8:45 – 9:15 | Ethical, scientific and regulatory principles governing the care and use of laboratory animals |
| 9:15 – 9:45 | Alternatives to laboratory animals |
| 9:45 – 10:15 | Principles of animal research methodology (1) |
| 10:15 – 10:45 | Break |
| 10:45 – 11:15 | Principles of animal research methodology (2) |
| 11:15 – 12:15 | Basic biology and behavior of common laboratory animal species |
| 12:15 – 12:45 | Animal husbandry and care |
| 12:45 – 13:15 | Safety in laboratory animal facilities |
| 13:15 – 14 | Lunch |
| 14 - 19 | Practical session- Group 1 |

| **Day 2** | |
| --- | --- |
| **Time** | **Topic** |
| 8:30 - 9:30 | Principles of sampling and drug administration |
| 9:30 – 10:15 | Recognition of pain and distress in laboratory animals |
| 10:15 – 10:45 | Break |
| 10:45 – 12:15 | Basic laboratory animal anesthesia and analgesia |
| 12:15 – 13:00 | Humane endpoints and euthanasia |
| 13:00 – 13:15 | Completing the post-workshop questionnaires |
| 13:15 – 14 | Lunch |
| 14 - 19 | Practical session- Group 2 |
