# Supplementary material for: A proposed framework for holding intensive 3Rs workshops in laboratory animal science
Source: Lab Anim Res. 2022 Mar 29;38:10. doi: 10.1186/s42826-022-00120-9 (PMC8966152; doi:10.1186/s42826-022-00120-9)
Supplement: Supplementary file 2 — Additional file 2: Ethical framework for holding intensive laboratory animal workshops. Proposed ethical framework is presented in this supplement. [file 42826_2022_120_MOESM2_ESM.docx]

Ethical Framework

for Holding Intensive Laboratory Animal Workshops

This framework describes the responsibilities of lecturers/supervisors and attendees, and defines the requirements of animals and facilities. In this text, ‘responsibilities’ are related to the person(s) having the ultimate responsibility for an action. ‘Requirements’ relate to the properties that an entity should possess to meet the scientific and ethical objectives of the workshop.

# Responsibilities of Lecturer and Supervisors

1. Teaching ethical aspects of working with laboratory animals should be prioritized to teaching mere scientific techniques. It is thus necessary to adhere to the principles of 3Rs as:
   1. Replace the laboratory animals, whenever possible,
   2. Reduce the number of animals used, when there is no replacement available,
   3. Refine the techniques so that they cause the least amount of pain and distress to animals.
2. Lecturers and supervisors should demonstrate role models of care and compassion toward animals.
3. The optimum number of attendees in practical sessions held by an experienced lecturer is 15-20 attendees. Attendees should be divided into groups of 5, with each group having a competent supervisor. Supervisors should have competency in ethics and scientific techniques of working with laboratory animals.
4. The workshop lecturer teaches the techniques, oversees the performance of the supervisors and attendees, and answers their questions.
5. The lecturer should deliver the required theoretical information for properly performing a technique, before demonstrating how to perform the technique.
6. The lecturer should only teach the techniques that are required for the competence of the attendees in performing their functions. If there are several techniques available to meet an educational objective, the technique which causes the least amount of pain and distress and is reasonably effective to accomplish the educational objective should be taught.
7. In introductory workshops, no dissection should be performed on live animals. Instead, animations and films should be used for teaching anatomy and dissection techniques.
8. Teaching surgery is not recommended in introductory workshops. A short session on surgery and a few hands-on practice could not deliver enough information and skills required for properly performing a surgical operation. Ironically, it may develop a false sense of confidence in the attendees for undertaking surgical procedures above their skills and capabilities.
9. An induction should be held at the beginning of the practical session, containing information about the safety of working with laboratory animals, equipment, and materials in the practical venue. Methods of prevention and management of animal bites and needle sticks should be taught.
10. Controlled materials (such as ketamine or opioids) and dangerous equipment should be in possession of the lecturer or supervisors and may be used by attendees only under supervision.
11. If an attendee reports dizziness, lethargy, lightheadedness, or other unusual symptoms, he/she should take a rest outside the practical venue. It is necessary to monitor his/her health and refer him/her to a physician if needed.
12. For training attendees who demonstrate unusual fear toward animals (e.g., phobia), only tranquilized animals should be used. These persons should be under the supervision of the most experienced supervisor in the venue, and the lecturer should pay more attention to their progress. If their fear do not subside after a while, their management in working with animals requires specialized psychological consultation, and it is necessary to avoid forcing them to touch or work with the animals during the workshop.
13. Before performing procedures that may cause bleeding or exposure of blood (or showing video clips containing such), it is necessary to remind attendees to avoid watching these scenes if they are sensitive to seeing blood (i.e., hemophobia).
14. Techniques that may cause pain or distress to animals must be demonstrated using videos, images, animations, or mannikins. If it is necessary to demonstrate some techniques on live animals, and these techniques have the potential to cause pain or distress, they should be only performed on properly anesthetized animals. Techniques that do not cause pain or distress can be demonstrated on conscious animals.
15. Before teaching the techniques on live animals, enough time should be given to attendees to familiarize themselves with the normal behaviors of the animals. During which time, a sense of compassion toward animals should be developed in attendees.
16. For practicing injections on live animals, blunt tip needles should be used. Only attendees that require performing injection on animals in their project may practice real injections under the supervision of group supervisors. The injections should be recorded in the lab record sheet, to avoid excessive number of injections on an animal, or accidental overdosing of animals by multiple injections.
17. In special occasions where a stock solution would be used for injection practices, aseptic rules of handling the stock solution should be taught first.
18. If a medication is planned to be injected, the attendees’ dose calculations should be double- checked by supervisors before giving the injection to animals.
19. When practicing tail vein access, at least one vein of the organ should remain intact so that it provides enough blood drainage for the organ. For example, attendees may be reminded to use either the left or right caudal vein and avoid using the dorsal vein. If an animal is intended to be used for another workshop, according to the conditions set forth in this ethical framework, the right caudal vein should be used in the first workshop, and the left caudal vein should be used in the second workshop
20. Attendees should be encouraged to give a treat to animals following the performance of a painful or stressful procedure. Commercial rodents’ and rabbits’ treats could be used for this purpose. Alternatively, a small piece of cheese, boiled egg, popcorn, meat, or boiled chicken bone may be used.
21. Supervisors should monitor the animals’ health and mood during the workshop. They should identify the signs of distress, fatigue, or lethargy in animals and avoid further use of the animals that are showing these signs. These animals should have rest and receive proper care.
22. Heart puncture should be taught using video clips. It should be reminded to attendees that heart puncture must only be performed on animals under surgical anesthesia. Following this procedure, the animal must be euthanized without gaining consciousness.
23. Blood sampling from retroorbital plexus of mice should be taught using video clips. It should be reminded to attendees that this is a terminal technique, the animal must be deeply anesthetized, and topical ophthalmic anesthesia (such as tetracaine) should be applied.
24. Methods of physical euthanasia (such as severing major blood vessels, cervical dislocation, decapitation) must not be taught on live animals. It should be emphasized to attendees that physical methods of euthanasia should be avoided as far as possible.
25. Due to the stressful environment of the practical session for laboratory rabbits, they may become agitated and struggle in the rabbit restrainers, leading to lumbar dislocation or pelvic fractures. Therefore, it is advisable to avoid using rabbit restrainers as far as possible. Instead, one can use chemical tranquilization (e.g., acepromazine 1 mg/kg) or tightly wrap the rabbit in a large towel for a brief period.

# Responsibilities of Attendees

1. Attendees should acknowledge the capacity of animals for feeling and sensing a wide range of conditions. They should endeavor to cause the least amount of negative feelings and sensations to animals.
2. Attendance in the practical session is only limited to those who need to work with laboratory animals; such as people who are currently using laboratory animals in a project or may need to use them in near future. This contrasts with attendance in the theoretical sessions, which is available to everyone interested in the subject matter.
3. Attendance in the practical session is conditional to successfully passing the theoretical session’s assessment.
4. If an attendee feels dizziness, lethargy, lightheadedness, or other unusual symptoms, he/she should inform the workshop lecturer as soon as possible.
5. Attendees must work under the supervision of the lecturer and supervisors during the whole course of the practical session. They must only practice the taught techniques and avoid performing any procedure out of the workshop curriculum.
6. Attendees should only practice with the species of animals that they might need to use in their research project.
7. Attendees should wear personal protective equipment (at least a mask, a clean lab coat, pair of gloves, and shoe covers) in the practical venue. They should avoid unnecessary movements in the practical venue and should move gently when required. Attendees should speak quietly and avoid teasing with animals or other attendees. Taking photos or videos in the practical venue is not allowed.
8. While gentle handling of animals is of utmost importance, the procedures on the animals should be finalized in the shortest time possible. Specifically, restraining the animals should be kept to a minimum.
9. Attendees with no prior experience in syringe handling or injections should practice on mannikins before practicing on animals.
10. Only those attendees that need to perform injections on live animals in their studies and require hands-on training, are allowed to perform injections on live animals in the workshop. For this purpose, 31G needle should be used for rodents, and the least possible volume of injectants should be injected. All the procedures should be performed under the direct supervision of a supervisor.
11. Attendees should employ aseptic techniques when performing injections on live animals.
12. Before injecting a drug to an animal, the dose of the drug should be calculated on the lab record sheet and the previous history of injections to the animal should be considered. This history may include the type, number, and volume of prior injections. Dose calculations should be double-checked with supervisors before giving the injection.
13. Animals that are unable to protect themselves in groups due to lethargy, sedation, or anesthesia, must not be placed in group cages.
14. If there is a remnant of blood on an animal’s body, it should be cleaned before returning the animal to its group.
15. Attendees must not mix animals from different cages together.
16. The surplus animals or those that are planned to be euthanized at the end of the workshop must receive the same care as the animals that are planned to be kept alive.

# Animals Requirements

1. Mice and rats are the most common species of animals used in biomedical research. Therefore, most of the workshop techniques and practices should be focused on these species.
2. An approximate number of 3-5 mice and 2-3 rats are suggested for a group of five attendees in a practical session. One rabbit could be used for demonstration of nonpainful procedures to a class of 15-20 attendees, subjected that only a maximum of 1-3 attendees need to perform supervised nonpainful procedures on the animal.
3. Laboratory animals should be acquired from valid sources so that the lecturer could ensure that they are not carrying zoonotic diseases. Animal bites and needle sticks should always be considered as probable events in the practical sessions.
4. It is preferable to use surplus animals for the practical session and avoid ordering new animals specifically for the workshop.
5. When using animals that had been previously used in another research:
   1. Previous history of the use of these animals in research should not cause any risk to the attendees’ health,
   2. The maximum severity score of the previous projects/procedures on the animals should be “mild” or “moderate” according to the content of the “Guideline for the Care and Use of Laboratory Animals,”
   3. The general welfare status of the animal, both physically and mentally, should have been recovered,
   4. The maximum severity score of the intended projects/procedures on the animals should be “mild,” “moderate,” or “non-recovery” according to the content of the “Guideline for the Care and Use of Laboratory Animals,”
   5. The reuse of the animal should be performed according to the recommendations of the designated veterinarian, which considers the clinical history and health status of the animal.
6. If the animals should be reused in another practical session, there should be at least 3 days (preferably one week) rest for the animals between the two sessions. The reuse of the animals should follow the rules set forth in the “Guideline for the care and use of laboratory animals.”
7. Animals should be moved to the practical venue an hour before starting the practical session, to adapt to the new environmental conditions. Animals should be kept in a quiet place with dim light and temperature relatively similar to their original holding place. The original cage of the animals should be used for their transport. Different species should be kept separately so that they could not see, hear, or smell each other.
8. During the practical session, only one species of laboratory animals should be on the benches at any given time. It is advisable to start the workshop on mice and then remove the mice from the practical venue and start working on rats.
9. Each animal should be uniquely marked before being used in the practical session. A lab record sheet should be assigned to each animal. The unique mark of the animal should be entered in the lab record sheet. Any activity performed on the animal should be recorded in the lab record sheet.
10. Animals that show signs of agitation during the practical session may harm themselves or workshop attendees. These animals should receive proper tranquilization (acepromazine; mice: 2-5 mg/kg SC; rats: 2.5 mg/kg IP; rabbit: 1 mg/kg IM). If the medication did not provide the required tranquilization, the animal should be placed in a separate cage and not be used in the workshop.
11. For every hour of the workshop, at least 10 minutes of rest should be given to animals. This time should be dedicated to the care of the animals, including feeding, and watering them and avoiding manipulating them for any reason.
12. Painful procedures could only be performed on properly anesthetized animals. These animals should receive proper analgesia for the post-procedural period.

*Suggested anesthetics include*:

- 1. Mice: ketamine (80-100 mg/kg; IP) + xylazine (10 mg/kg; IP) + acepromazine (3 mg/kg; IP);
  2. Rats: ketamine (40-50 mg/kg; IP) + xylazine (2-10 mg/kg; IP) + acepromazine (0.5-1.5 mg/kg; IP).

*Suggested analgesics include:*

- 1. Buprenorphine
     1. Mice: 0.05-0.1 mg/kg; SC
     2. Rats: 0.01-0.05 mg/kg; SC or 0.1-0.25 mg/kg PO

AND

- 1. Topical application of lidocaine-prilocaine ointment on the skin at the practice site (if applicable).

AND, either

- 1. meloxicam:
     1. Mice: 5 mg/kg; SC
     2. Rats: 1 mg/kg; SC

OR

- 1. Ketoprofen:
     1. Mice: 5 mg/kg; SC
     2. Rats: 5 mg/kg; SC

1. If it is planned to anesthetize animals during the workshop, the timing should be planned such that the depth of anesthesia is reduced before the end of the workshop and the animal should be able to hold sternal position. Anesthetized animals should not be left unattended at the end of the workshop.

# Facilities Requirements

1. The practical venue should be in a quiet place of the building.
2. The floor and walls of the practical venue should be washable.
3. There should be proper ventilation available in the practical venue.
4. The temperature of the practical venue should be adjusted between 20-24 °C.
5. There should be at least four relatively large benches in the practical venue so that each group of 5 attendees could use one bench for their practices.
6. All benches and chairs in the practical venue should be washable. They should be washed before and after the practical session.
7. The benches should be covered with paper covers or large thick plastic bags.
8. There should be a video projector and a screen in the practical venue so that the lecturer could deliver the theoretical matters related to each part of the practical session.
9. There should be a whiteboard available in the practical venue for teaching dose calculation and drug dilution.
10. Warm water (38 °C) should be available during the practical session for facilitating tail vein access on mice and rats.
11. Following completion of teaching on one species, bench covers should be collected. The benchtop and the used equipment should be wiped with 3% hydrogen peroxide solution to remove probable pheromones of rodents. The practical venue should be ventilated properly, and tables should be covered with new coverings. Then a new species of animals can be brought into the venue.
