# Supplementary material for: A proposed framework for holding intensive 3Rs workshops in laboratory animal science
Source: Lab Anim Res. 2022 Mar 29;38:10. doi: 10.1186/s42826-022-00120-9 (PMC8966152; doi:10.1186/s42826-022-00120-9)
Supplement: Supplementary file 3 — Additional file 3: Lab record sheet. A sample of the lab record sheet is presented in this supplement. [file 42826_2022_120_MOESM3_ESM.docx]

**Lab record sheet**

| Full name of the responsible person: | | | | | | | | | |
| --- | --- | --- | --- | --- | --- | --- | --- | --- | --- |
| Animal species: | | | Animal No.: | | | | Weight: | | |
| Animal identification: | | | | | | | | | |
| Animal sex: | | | | | | | | | |
| **Injections**  **(Only with prior arrangement with the workshop lecturer and under supervision)** | | | | | | | | | |
| For each injection, tick a number and the corresponding empty box in front of it. Maximum of four injections (regardless of the type) are allowed. | | | | | | | | | |
| **Number of injections** | | **1** | | **2** | | **3** | | | **4** |
| Subcutaneous injection | |  | |  | |  | | |  |
| Intraperitoneal injection | |  | |  | |  | | |  |
| Venous access | |  | |  | |  | | |  |
| Intramuscular injection | |  | |  | |  | | |  |
| **Details of the injections performed on the animal** | | | | | | | | | |
| Medication | Dose | | | | Injection Volume | | | Injection route | |
|  |  | | | |  | | |  | |
|  |  | | | |  | | |  | |
|  |  | | | |  | | |  | |
