# Supplementary material for: A proposed framework for holding intensive 3Rs workshops in laboratory animal science
Source: Lab Anim Res. 2022 Mar 29;38:10. doi: 10.1186/s42826-022-00120-9 (PMC8966152; doi:10.1186/s42826-022-00120-9)
Supplement: Supplementary file 4 — Additional file 4: Practical session structure. The structure of the practical session is presented in this supplement. [file 42826_2022_120_MOESM4_ESM.docx]

**Practical session structure**

## 1. Induction and grouping

An induction is held in the beginning of the practical session and attendees are familiarized with the practical venue and safety measures of working with animals. The ethical rules of the workshop pertinent to the attendees (according to the ethical framework discussed in Supplement 2), are described to the attendees. The aim and overall plan of the practical session are defined.

## 2. Topics in practical session

Mice (Mus musculus) are the first animal species to teach, with five mice being allocated to each group of five attendees. Topics are taught according to the order of the topics presented in Table 1 of this paper. Following completion of the mice techniques, they are removed from the practical venue and certain preparations are done according to the ethical framework (Supplement 2). Then, rats (Rattus rattus) are brought into the practical venue and three rats are assigned to each group. After removing rats, a rabbit is brought into the practical venue. Maximum 3 attendees are allowed to work with the rabbit under supervision of the workshop lecturer, if they: 1) need to perform certain interventions on rabbits in their ongoing research or any research within one year after completion of the workshop, and 2) The actual severity score of the intended procedure is “mild”.

### Normal behaviors of animals

Normal behaviors of each animal species are described by photos, videos, and demonstration. Time is given to attendees to become familiar with animals without performing any intervention on them. Attendees are allowed to gradually touch the animals, pet them, provide them with treats, hold them in hands (without restraining), as long as their action is not distressing the animals within the discretion of the group supervisor. By using laboratory grown animals that are used to handling, this practice rarely causes distress to them.

### Animals marking

Attendees assign a number to each animal and mark the animal by writing the number on the base of its tail and allocate one lab record sheet to each animal.

### Animal handling, sexing, restraining, and heterospecific play

Various methods of handling, sexing, and restraining the animals are taught using video clips. Heterospecific play with rats (rat tickling) and its welfare benefits [1, 2] are presented. Then the attendees perform these techniques under the supervision of group supervisors.

### Giving treats and rest to animals

Treats are given to animals, and they are left undisturbed to rest in their original cages for 10 minutes.

### Dose calculation and discussion of the effects of selected medications

Methods of dose calculation, stock solution preparation, and making various dilutions are taught. Theoretical and practical aspects of the use of acepromazine (tranquilization: 2mg/kg, SC and sedation: 5mg/kg, SC), ketamine + xylazine (100mg/kg + 10mg/kg, IP), ketoprofen (5mg/kg, SC), and tramadol (15mg/kg, SC) are discussed.

### Syringe handling

Syringe and needle handling techniques, such as scoop technique and precision injections, are taught. The coordinated action between the syringe handling hand and the animal restraining hand is taught using silicone egg shaped grip balls, as a replacement to animals.

### Subcutaneous, intraperitoneal, and intramuscular injections

Subcutaneous, intraperitoneal, and intramuscular injection techniques are taught using video clips and demonstration by blunt-tip needles without penetrating the skin of the animal. Attendees are then given time to practice injection techniques on mannikins and then animals using the blunt-point needles. Only those attendees who need to practice real injection on animals (at the discretion of the workshop lecturer), are allowed to do so under direct supervision of the workshop lecturer. The 31-gauge needles are used for this purpose.

### Oral gavage

Oral gavage techniques using rigid and flexible canula are taught by slide presentation.

### Venous access

Venous access techniques in mice and rats (tail vein, saphenous vein, retroorbital plexus, and abdominal vessels) and rabbits (marginal ear vein, central ear artery) are taught using slide presentation. Attendees are then encouraged to use injection training pads to practice venous access, as an alternative to using animals. These injection pads may be obtained commercially or be made for the workshop (Fig. 1).

For making these injection pads, solution of water and a detergent (e.g., dishwashing liquid) is prepared. A small volume of silicon sealant is poured into the water. The silicone sealant is mashed by gloved hands.A rectangular plastic cast wetted with the same water solution is used to mold the silicone sealant. Copper wires of various dimeters are passed through the molded silicone sealant. The mold is put in the room temperature for 24 hours to harden. Afterwards, the wires are pulled out, creating hollow structures resembling vessels. A water-soluble color (e.g., red poster paint) is used to make a blood-like fluid. The fluid is injected into the vessel-like structures using a vascular catheter and a syringe (Fig. 1).


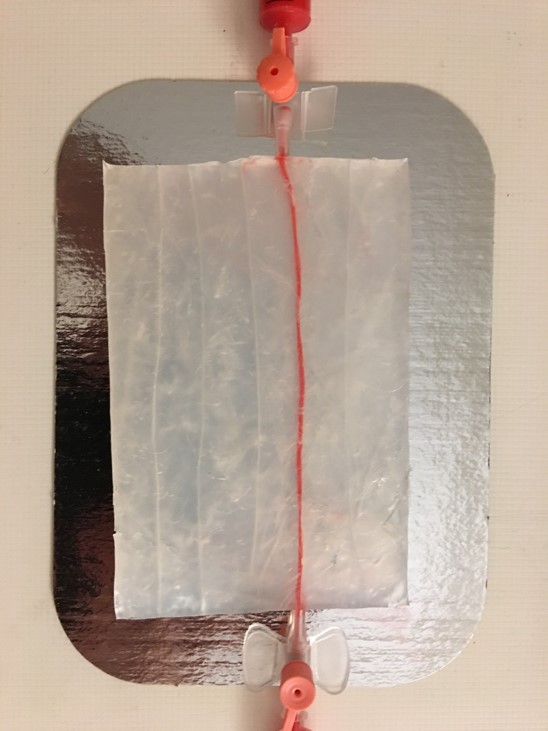


Fig. 1) A handmade injection pad with vessel-like structures. A blood-like fluid is used to simulate a blood filled vessel.

### Management of anesthesia and analgesia

Simple techniques for managing anesthetized animals are taught. These include hypothermia prevention, eye lubrication, respiratory assessment, assessment of the depth of anesthesia and analgesia, and hypoglycemia prevention

### Medication assignment and administration

The anesthetic protocol comprising either ketamine + xylazine (100 mg/kg + 10 mg/kg, IP) and ketoprofen (5 mg/kg, SC) or ketamine (100 mg/kg; IP) + xylazine (10 mg/kg; IP) + acepromazine (3 mg/kg; IP) is assigned to the groups. Each group is asked to weigh one or two mice, calculate the dosage of the above medications, and administer the medications to the mice under supervision of their supervisors.

### Tail vein access

Those attendees who essentially need to practice tail vein access are allowed to perform supervised practice for intravenous injection of 0.06 ml normal Saline (maximum 4 times) or 0.04 ml blood collection (maximum 3 times). The mice are checked to be in the surgical plane of anesthesia. Lidocaine (10%) is applied to the tail before attempting vascular access. Two attempts are allowed for each vein.

## 3. Workshop termination

At the end of the workshop, the animals are managed according to the institutional rules and regulations regarding the animals used for teaching.

**References**

1. LaFollette MR, O’Haire ME, Cloutier S, Gaskill BN. Practical rat tickling: Determining an efficient and effective dosage of heterospecific play. Applied Animal Behaviour Science. 2018;208:82–91.

2. LaFollette MR, Cloutier S, Brady C, Gaskill BN, O’Haire ME. Laboratory animal welfare and human attitudes: A cross-sectional survey on heterospecific play or “rat tickling.” PLOS ONE. 2019;14:e0220580.
